# Fabrication of Tissue-Engineered Cartilage Using Decellularized Scaffolds and Chondrocytes

**DOI:** 10.3390/polym14142848

**Published:** 2022-07-13

**Authors:** Liang Lu, Xifu Shang, Bin Liu, Weijian Chen, Yu Zhang, Shuyun Liu, Xiang Sui, Aiyuan Wang, Quanyi Guo

**Affiliations:** 1 Department of Orthopaedics, The First Affiliated Hospital of China University of Science and Technology, Hefei 230026, China; luliangzhwk@163.com (L.L.); shangxifu@163.com (X.S.); binde238@163.com (B.L.); chenweijian2007@163.com (W.C.); 2 Sports Medicine and Adult Reconstructive Surgery, Nanjing Drum Tower Hospital, Nanjing 210008, China; diebiyou4657@163.com; 3 Institute of Orthopaedics, 301 Hospital of the Chinese People’s Liberation Army General Hospital, Beijing 100120, China; clear_ann@163.com (S.L.); suixiang301@126.com (X.S.); wangaiyuan301@126.com (A.W.)

**Keywords:** tissue engineering, scaffolds, chondrocytes, articular cartilage, decellularized

## Abstract

In this paper, we aim to explore the application value of tissue engineering for the construction of artificial cartilage in vitro. Chondrocytes from healthy porcine articular cartilage tissue were seeded on articular cartilage extracellular matrix (ACECM) scaffolds and cultivated. Type II collagen immunofluorescent staining was used to assess secretion from the extracellular matrix. Chondrocytes, which were mainly polygonal and cobblestone-shaped, were inoculated on ACECM-oriented scaffolding for 7 days, and the neo-tissue showed translucent shape and toughness. Using inverted and fluorescence microscopy, we found that chondrocytes on the scaffolds performed well in terms of adhesion and growth, and they secreted collagen type II. Moreover, the porcine ACECM scaffolds had good biocompatibility. The inflammatory cell detection, cellular immune response assay and humoral immune response assay showed porcine ACECM scaffolds were used for xenotransplantation without significant immune inflammatory response. All these findings reveal that ACECM-oriented scaffold is an ideal natural biomaterial for cartilage tissue engineering.

## 1. Introduction

Articular cartilage (AC) has no blood vessels, nerves, or lymphatic systems. The main component of AC is the extracellular matrix (ECM), with a sparse population of chondrocytes distributed throughout the tissue [1]. Because of the absence of blood vessels, undifferentiated stem cells are absent in the AC, and chondrocytes are confined to dense pits of collagen and proteoglycans [2]. Despite advances in technology and biotechnology, local cartilage defects of the knee are common and continue to present clinical challenges. These patients are often young, physically active individuals who may also be affected by ligament, meniscus or soft tissue damage, further complicating treatment [3]. Currently, although patients with early AC injury can be treated conservatively and surgically, conservative treatment can only reduce the swelling and local pain around the joint. This approach is unable to cure the AC damage. In terms of surgical methods, the mechanical outcome is poor and the lack of abrasion resistance eventually leads to regression [4]. Moreover, the repair of cartilage defects through the transplantation of periosteum, bone perichondrium, and osteochondral material to try to produce hyaline cartilage has been attempted [5,6]. However, results of long-term clinical results for patients are uncertain. Thus, research on the repair of cartilage defects is urgently needed.

Tissue engineering technology [7,8], in which the principles and methods of engineering and life sciences are applied to prepare bioactive cell-scaffold complexes in vitro, has been explored to repair damaged tissues [9]. Remarkable achievements have been made in cartilage tissue engineering. For example, Rotter et al. [10] planted human cartilage tissue onto a mixture of polylactic acid and poly(glycolic acid) and transplanted it into mice to successfully form hyaline cartilage matrices with stable mechanical properties. Jacob et al. [11] revealed that a smart piezoelectric nanohybrid of poly(3-hydroxybutyrate-co-3-hydroxyvalerate) and barium titanate could stimulate cartilage regeneration; moreover, the piezoelectric scaffolds can act as sensitive mechanoelectrical transduction systems, and this is applicable to regions where mechanical loads are predominant [12]. Actually, to date, a variety of materials have been used for different structures, including PLGA [13], agarose [14], chitosan [15], alginate [16], and collagen [17]. However, their mechanical properties are generally inferior to those of native cartilage. Thus, biomaterial scaffold-seeding chondrocytes that can provide biosynthetic activity both in vitro and in vivo are required.

In the last few years, an alternative technology using natural cell-free ECM biomaterials has obtained great attention in cartilage regeneration [18]. However, on the one hand, the decellularized cartilage ECMs were constructed based on different animal origin and different decellularization methods (e.g., chemical, physical and enzymatic) and agents. For example, Goldberg-Bockhorn et al. [19] used a custom-made glass bioreactor to enhance cell migration into decellularized porcine cartilage scaffolds and mimic physiological conditions. Gong et al. [20] developed decellularized cartilage ECM (dcECM) hydrogels from porcine ears innovatively via the main method of enzymatic digestion and verified good biocompatible properties of dcECM hydrogels to deliver chondrocytes and form subcutaneous cartilage. The 3D hierarchical porous BC/dcECM scaffolds with structurally and biochemically biomimetic cartilage regeneration microenvironment were fabricated by freeze-drying technique, which could exhibit excellent mechanical properties, water superabsorbency and shape-memory properties [20]. However, to date, the research related to safety analysis (such as immunoreaction) of articular cartilage derived extracellular matrix (ACECM) scaffolds has been limited. In the present study, autologous pig chondrocytes were cultured and inoculated on articular cartilage extracellular matrix (ACECM) scaffolds to construct tissue-engineered artificial cartilage in vitro and to test whether devitalized cartilage ECM can induce chondrogenic differentiation. Moreover, inflammatory cell detection, cellular immune response assay and humoral immune response assay was conducted based on the heterogenous subcutaneous implantation of ACECM scaffolds. The findings are expected to provide a theoretical basis for the application of porcine articular cartilage in tissue engineering.

## 2. Materials and Methods

### 2.1. Animals and Samples

Limbs were obtained from 13-month-old pigs soon after slaughter at a slaughterhouse in Beijing, China. Adult miniature pigs (approximately 20 kg) and New Zealand white rabbits (approximately 2.5 kg) were obtained from the experimental animal center of the Peoples Liberation Army General Hospital (Beijing, China). The study protocol was approved by the Ethics committee of the People’s Liberation Army General Hospital: 2009-x01-15, 2009-07-09.

### 2.2. Preparation of ACECM Scaffolds

**Figure 1:**
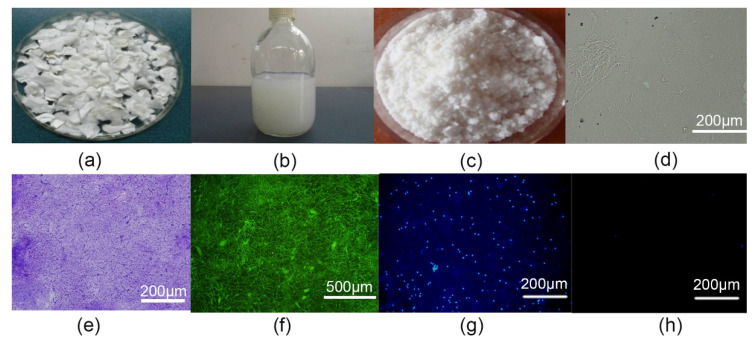
(**a**) Fresh articular cartilage. (**b**) Articular cartilage extracellular matrix (ACECM) slurry with a milky white appearance. (**c**) ACECM dry powder. (**d**) Micro-filament structure of ACECM slurry. (**e**) ACECM smear for toluidine blue staining. (**f**) Positive ACECM smear for collagen type II immunohistochemical staining. (**g**) ACECM slurry smear for Hoechst33258 fluorescence staining before differential centrifugation (light microscopy, ×100 magnification). (**h**) ACECM slurry smear examined by Hoechst33258 fluorescence staining after differential centrifugation (light microscope, ×100 magnification).

The preparation method was based on the previous study [21]. Briefly, the AC of pig limbs (Figure 1a) was soaked in phosphate buffered saline (PBS; Sigma-Aldrich, St. Louis, MO, USA) containing phenylmethylsulfonyl fluoride (Sigma-Aldrich, St. Louis, MO, USA) at 0 to 4 °C to inhibit some proteases and prevent proteases from breaking down the collagen components in cartilage tissue, which would result in the loss of important components of the extracellular matrix of cartilage. Then, tissues were mashed by ultramicro wet grinding method. After dilution, the suspension was differentially centrifuged at 4 °C as follows: The slurry was centrifuged at 4 °C and freeze-dried. The diluted solution of the powder, which was prepared at a concentration of 2%, was injected into a polyethylene cylinder mold. A freeze-drying stent was irradiated with ultraviolet light at 258 nm and crosslinked with an anhydrous ethanol solution of carbonimide (Sigma-Aldrich, St. Louis, MO, USA) and *N*-hydroxysuccinimide (Sigma-Aldrich, St. Louis, MO, USA). The crosslinked material was washed with disodium hydrogen phosphate for 2 h, soaked in aseptic PBS for 2 h, rinsed 3 times with steam, sterilized, and sealed for later use.

### 2.3. Observation of ACECM and Decellularized Cartilage Scaffold

The freeze-dried powder was dissolved in distilled water to prepare a 0.5% suspension. After conventional smear preparation, the composition of the slurry was qualitatively analyzed by toluidine blue (Sigma-Aldrich, St. Louis, MO, USA) and collagen type II (Sigma-Aldrich, St. Louis, MO, USA) staining. Hoechst 33258 fluorescent dye (Sigma-Aldrich, St. Louis, MO, USA) was used to detect the cell residues. The sizes of the fibers in the slurry were observed by scanning electron microscopy (SEM; Olympus BX51, Japan). The scaffold was fixed in 10% neutral formalin for 12 h, dehydrated with gradient alcohol, embedded in paraffin, and sectioned into 5 μm-thick sections. These were used for histological and immunohistochemical staining.

### 2.4. Biological Safety Analysis of ACECM Scaffold

For hemolysis test, 2 mL peripheral blood of healthy person was collected and diluted with 2.5 mL 0.9% NaCL. Then, the experiment was divided into three groups: experimental group (scaffold extract, mixed with 0.9% NaCL and ACECM scaffold surface area at a ratio of 1 mL: (3~6) cm^2^), positive control group (double steamed water) and negative control group (normal saline). After 0.2 mL of peripheral blood was added to each group, the A value was measured at 545 nm wavelength, and the degree of hemolysis was calculated according to the following formula: Hemolysis degree = (experimental group A value − negative control group A value)/(positive control group A value − negative control group A value) × 100%. The degree of hemolysis < 5% was considered as no hemolysis.

The surface area of serum-free DMEM culture medium (HyClone, GE Healthcare, Little Chalfont, UK) and acellular cartilage scaffold was mixed at the ratio of 1 mL: (3–6) cm^2^ and stood at 37 °C for 72 h to obtain the scaffold extract. Based on scaffold extract, pyrogen assay was conducted using the endotoxin detection kit, following the manufacturer’s instructions.

For the intradermal test, 5 points were selected on each side of the spine (2 points 2 cm apart) of three New Zealand white rabbits. Next, 0.2 mL scaffold extract was injected intradermal at each point on one side, and as control, the same amount of normal saline was injected intradermal at each point on the other side. The reactions of each injection site and surrounding tissues were observed at 0, 24, 48 and 72 h after injection.

### 2.5. Isolation and Culture of Porcine AC Chondrocytes

Porcine AC was rinsed several times with Hank’s liquid and cut into 1 mm^3^ samples. Collagenase type II (0.15%; Abcam, Cambridge, MA, USA) was added for digestion and stirred for 1 h at 37 °C until the digestive juices become cloudy. The turbid digestive fluid was centrifuged at a speed of 1000 rpm for 5 min and washed with Hank’s liquid. The chondrocyte culture solution containing 20% fetal bovine serum (HyClone, Logan, UT, USA) was added to dissolve the cell masses, and chondrocyte suspension was then transferred to 10 mL ordinary culture flask with nylon gauze and cultured in an incubator at 37 °C in an atmosphere of 5% CO_2_. All the procedures were repeated three to four times until the cartilage was completely digested. For cell activity identification, a drop of digested chondrocyte suspension was mixed with acridine orange-propidium iodide (AO-PI) dye and observed under the excitation light wavelength of 450~490 nm.

Adherent cells were selected for further expansion. The cells were inoculated on a cover glass positioned in each well of a 6-well culture plate. When cell growth was 80–90% confluent, the cover glass containing the cells was removed from the well, and the cells were fixed with a 1:3 solution of glacial acetic acid and methanol for 10 min, followed by Giemsa staining and fixation with 4% paraformaldehyde for more than 5 min. Then, the prepared cell slides were stained with safranin “O” (Sigma-Aldrich, St. Louis, MO, USA), toluidine blue, and collagen type II. Briefly, for safranin “O” staining, the fixed cell slides were rinsed with PBS three times and then stained with hematoxylin for 3 min. After differentiation, they were washed with water to return to a blue color, followed by 3% bright green staining for 5 min. For toluidine blue staining, fixed cell slides were dried and dyed with freshly prepared dye for 10 min. Then, the alcohol dehydrate, xylene transparent, resin seal, and light microscopy observations were conducted. For collagen type II, fixed cell slides were with 3% H_2_O_2_/methanol at room temperature for 10 min to eliminate endogenous peroxidase activity. After blocked with 10% normal rat serum (diluted with PBS) for 10 min, the rat anti-human type II collagen polyclonal antibody was added, followed by labeled secondary antibody at 37 °C for 30 min and horseradish enzyme labeled ovalbumin at 37 °C for 30 min. In the end, samples treated in accordance with the aforementioned method were observed under a light microscope.

### 2.6. Inoculation of Porcine Articular Chondrocytes on Scaffolds

The third-generation chondrocytes were centrifuged at 1500 r/min for 5 min, the supernatant was discarded, and the DMEM culture medium (HEPES, 2.97 g; L-proline, 0.046 g; vitamin C, 0.05 g; ITS, 10 mL; NEAA, 10 mL) was removed to prepare the cell suspension at a concentration of 1 × 10^7^/mL. In order to dynamically observe the morphology, adhesion, proliferation, distribution and arrangement of cells on the scaffold, the chondrocytes were labeled with PKH26 fluorescent dye according to the product instructions, and the labeled cells were prepared into 1 × 10^7^ cells/mL cell suspension for subsequent use.

Cell suspensions (1 × 10^7^ cells) were injected into the interior of the ACECM scaffolds. Subsequently, the cell-scaffold complex was transferred to an incubator at 37 °C in a 5% CO_2_ atmosphere for 2 h. During this period, chondrocyte culture (10 μL) was added every 30 min, and the scaffolds were flipped. The chondrocyte culture medium (5 mL) was then slowly added to each well. The medium was changed every other day.

### 2.7. Examination of Cell–Scaffold Complexes

Gross observations included morphology, color, texture, and volume changes. Morphological observation was performed after 1 d of culture. The unlabeled cell–scaffold complex was stained with Hoechst33258 for observation. Cell activity was detected by AO-PI staining after 1 week of culture via observation with a fluorescence microscope. Dead cells were red and living cells were green under the excitation light wavelength of 450~490 nm.

Moreover, the morphology, adhesion, proliferation, and distribution of chondrocytes on the scaffold were observed using an inverted microscopy. The morphology, adhesion, proliferation, and distribution of chondrocytes on the scaffold were observed using inverted microscopy and inverted fluorescence microscopy at 2 h, 1 d, 1 week (7 d), and 2 weeks (14 d) after inoculation. SEM was performed to evaluate the morphology, adhesion, proliferation, and distribution of chondrocytes cultured on the scaffolds after a 3-day cultivation. Immunofluorescence staining of collagen type II was observed by fluorescence microscopy.

### 2.8. Analysis of Immunoreaction of ACECM Scaffolds

For the preparation of non-acellular cartilage scaffolds, cartilage was cut from the joints of pig limbs without perichondrium and subchondral bone. After the cartilage was washed with normal saline and drained, it was cut into thin slices and pulverized. Then, 25–38 μm particles were sieved and dissolved in tri-distilled water, and the prepared solution was injected into a polyethylene cylinder mold. After freeze drying, the non-decellularized cartilage was prepared [22], and then stored at 4 °C for later use.

Then, the rabbits were divided into two groups for subcutaneous implantation experiment: decellularized cartilage scaffold group (n = 3) and non-decellularized cartilage scaffold group (n = 3). The decellularized cartilage scaffold was embedded between the deep fascia and sarcosis in the back of each rabbit (see Figure S1 in Supporting Information). Briefly, after anesthesia, rabbit’s back was routinely skinned, disinfected and covered, and incisions were made on both sides of the spine. The skin and subcutaneous tissue were cut in turn, and the cartilage scaffold was embedded between the deep fascia and sarcosis. Three embedding points were designed on the back of each rabbit, with the incision length about 1.5 cm and a distance of about 5 cm between each embedding point. All skin incisions were sutured with 3-0 fine silk thread. Observation was performed at 1, 2 and 4 weeks after implantation.

Subsequently, in order to determine the possible toxic reactions and other adverse reactions of experimental animals after implantation of each scaffold, the activities, diet and water intake of experimental animals were observed 1, 2 and 4 weeks after the operation, respectively. Moreover, inflammatory cell detection, cellular immune response assay and humoral immune response assay were performed, respectively. Briefly, for inflammatory cell assay, the tissue was frozen and sectioned with a thickness of 5µm, and stained with HE (Sigma-Aldrich, St. Louis, MO, USA). Immunohistochemical staining of cellular immune response assay was performed with monoclonal antibodies against rabbit T lymphocyte CD4 (1:200) and CD8 (1:200) and labeled with fluorescein reagent. For humoral immune response detection, peripheral blood of animals was extracted to detect the antibody concentration to scaffolds. After protein quantification, the cartilage matrix of the two scaffolds was diluted to 5 µg/mL by coating solution, and then added into 96-well plates, and sealed with 10% bovine innocence protein at 37 °C for 1 h. Then, experimental animal serum before surgery and 1, 2 and 4 weeks after surgery was added, respectively. After cleaning, biotinylated anti-mouse (rabbit) secondary antibody IgG (Sigma-Aldrich, St. Louis, MO, USA) was added at 37 °C. After washing, tri-antibody (chain ovalbumin) (37 °C; 0.5–1 h), and chromogenic agents (o-phenylenediamine and H_2_O_2_ dissolved in PBS) were successively added. The concentration was measured using a microplate reader at 490 nm.

### 2.9. Statistical Analysis

Statistical analysis was performed using the SPSS 13.0 software package (IBM SPSS Statistics, Chicago, IL, USA). All values were expressed as the mean ± standard deviation. Differences between two groups were analyzed by *t*-test. Values of *p* < 0.05 were considered statistically significant.

## 3. Results

### 3.1. Characterization of ECM Slurry of AC

After crushing, differential centrifugation, decontamination, and nucleation, the ECM of AC presented as a milky solution (Figure 1b) and formed a white powder after freeze-drying (Figure 1c). The smear of the ECM cartilage appeared small and fibrous with a uniform distribution and no cell structure when examined by light microscopy (Figure 1d). All samples stained positive for toluidine blue (Figure 1e) and collagen type II (Figure 1f). Staining with Hochest33258 showed the retention of cells in the slurry prior to differential centrifugation (Figure 1g). The ECM materials collected after differential centrifugation and ribosomal digestion showed no nucleation (Figure 1h).

### 3.2. Characterization of Scaffolds

**Figure 2:**
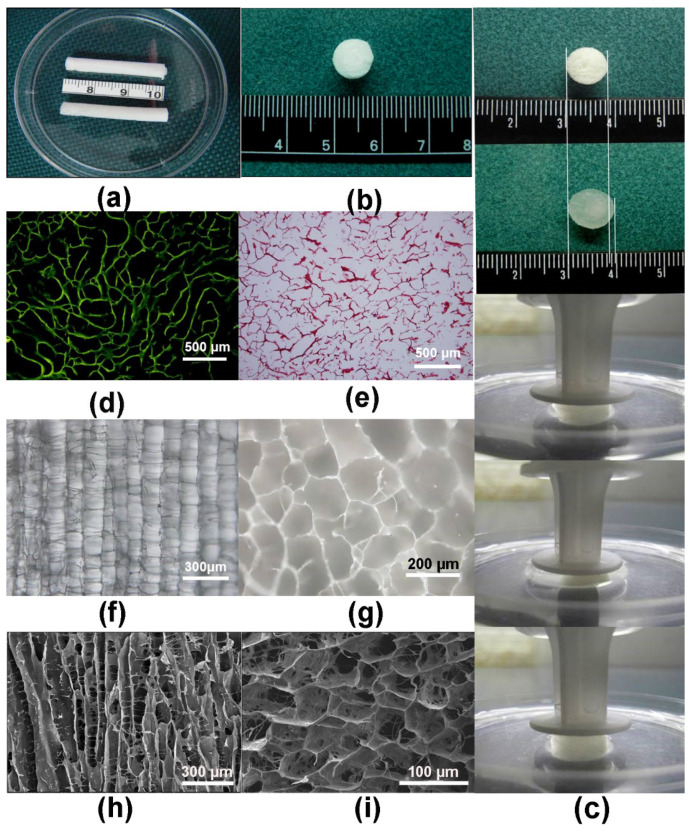
(**a**) General observation of scaffold. (**b**) Dry scaffold. (**c**) Comparison between dry and wet state scaffold. (**d**) Scaffold cross-section using collagen type II immunohistochemistry (light microscopy, ×100 magnification). (**e**) Safranin O staining of scaffold cross-section (light microscopy, ×100 magnification). (**f**) Longitudinal section of scaffold (light microscopy, ×100 magnification). (**g**) Scaffold cross-section (light microscopy, ×100 magnification). (**h**) Scaffold longitudinal section (light microscopy, ×130 magnification). (**i**) Scaffold cross-section (light microscopy, ×130 magnification).

As shown in Figure 2a, the general shape of the scaffold could be prepared according to different shapes of the molds. A tubular structure was faintly apparent on the scaffold. The comparison between the dry and wet states indicated that the scaffold had certain water absorption expansibility and compression extensibility (Figure 2b,c). Both samples stained positive for collagen type II (Figure 2d) and safranin O (Figure 2e), indicating that the basic components of the scaffold were consistent with those of the ECM of AC and extracted ECM materials. An examination of longitudinal sections of the channel revealed a pipe structure with a parallel arrangement (Figure 2f,h), which showed a uniform distribution of holes and regular shapes. An examination using light microscopy and SEM showed that the cross-section of the stent had a porous honeycomb structure (Figure 2g,i).

**Figure 3:**
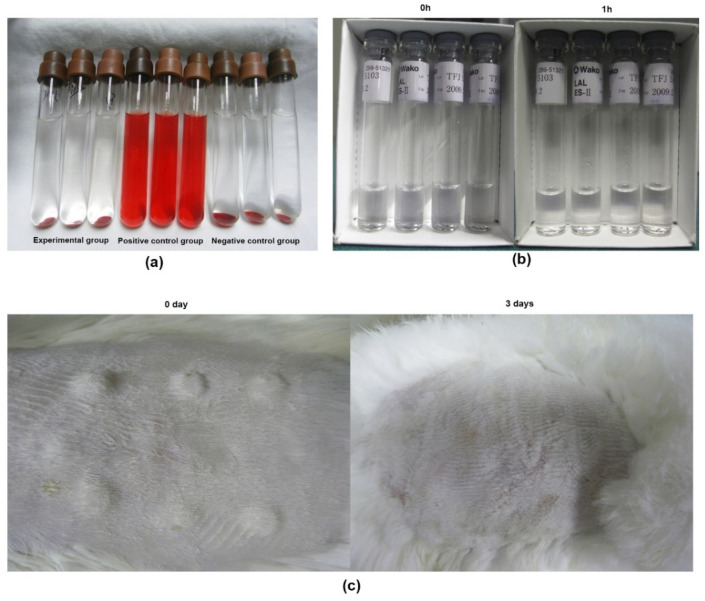
The biological safety of scaffolds. (**a**) The hemolysis test in the experimental group, negative control group, and positive control group. (**b**) The pyrogen test of scaffold extract. (**c**) The intradermal test of scaffold extract on rabbit.

The hemolysis test of biological safety of scaffolds showed there was no obvious hemolysis in the experimental group and the negative control group, and hemolysis in the positive control group (Figure 3a). Furthermore, the pyrogen test of scaffold extract was negative, indicating that no pyrogen existed (Figure 3b). After subcutaneous injection of scaffold extract, no erythema, edema and other stimulating reactions were found at each injection site and its surrounding tissues (Figure 3c).

### 3.3. Characterization of Chondrocytes

Adult miniature pig knee cartilage was collected and digested with collagen type II at a mass fraction of 0.15%. The activity of the cells was identified using AO-PI dye; over 90% of the cells were viable (see details in Figure S2 of Supporting Information). The primary cells were cultured in the culture medium. Generally, a few cells were attached to the wall after 6–8 d. The rounded cells were enlarged and elongated to form protuberances and were spread out on the culture dish. The newly attached cells were short fusiform or polygonal in shape. With an increase in incubation time, the cells attached to the square hole in the gauze and gradually grew to the center of the hole and fused. Deposition of the surrounding matrix caused growth of the cell layers and the formation of tissue masses. Third-generation chondrocytes generally adhered to the wall for approximately 10 h, after which, they spread rapidly to form small polygons (see details in Figure S3 of Supporting Information). Moreover, the cells stained positive for safranin O, collagen type II, and toluidine blue.

### 3.4. Characterization and Biological Safety of ACECM-Derived Scaffolds

**Figure 4:**
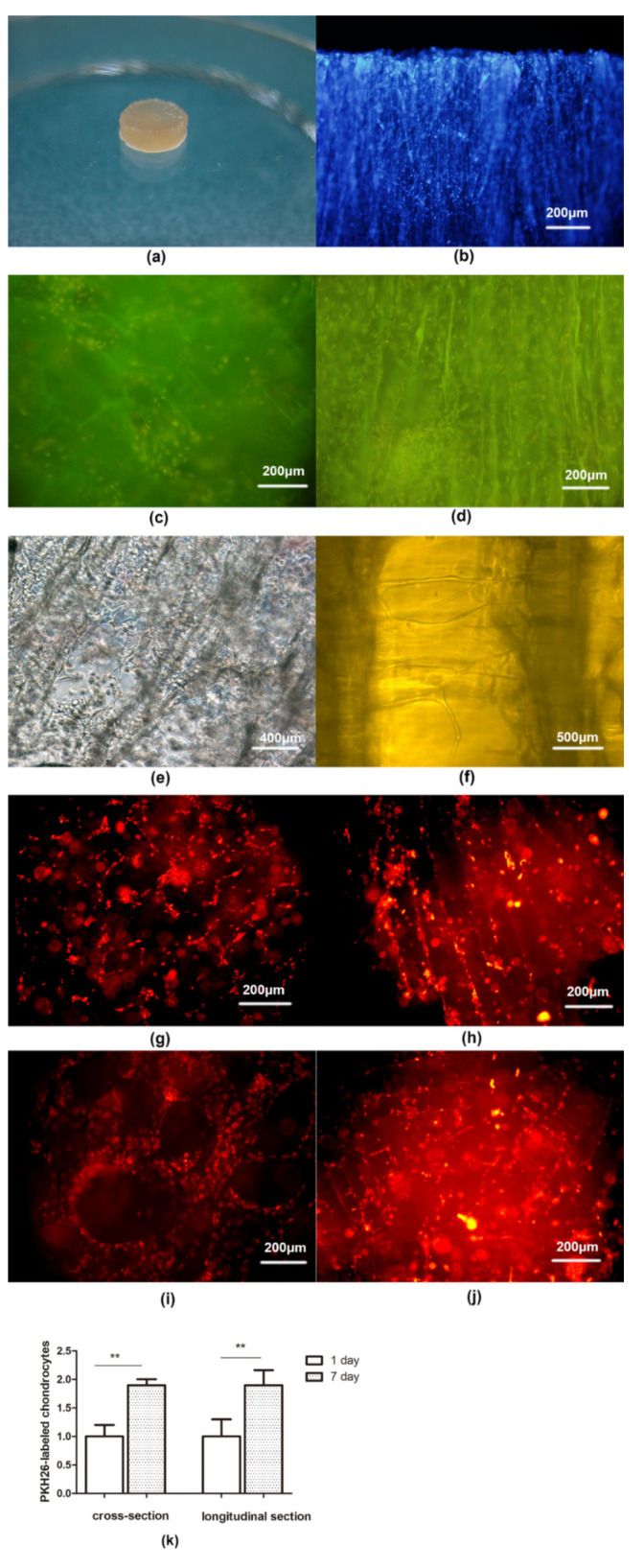
(**a**) After 1 week (7 d) of in vitro culture, the cells were combined into the scaffold. (**b**) Distribution of pig chondrocytes (Hoechst 33258 labeled nuclei) on the scaffolds (inverted fluorescence microscopy, ×100 magnification). (**c**) Fluorescence microscopy of acridine orange-propidium iodide (AO-PI)-stained sample after 24 h inoculation (cross-section, ×100 magnification). (**d**) Fluorescence microscopy of AO-PI-stained sample after 24 h inoculation (longitudinal section, ×100 magnification). (**e**) Chondrocytes were inoculated on the scaffold for 2 h (inverted microscopy, ×200 magnification). (**f**) Adhesion and proliferation of chondrocyte composite scaffolds after 1 week of in vitro culture (inverted microscopy, ×400 magnification). (**g**) PKH26-labeled chondrocytes on the scaffold cultured for 1 d (cross-section, inverted fluorescence microscopy, ×40 magnification). (**h**) PKH26-labeled chondrocytes on scaffolds cultured for 1 d (longitudinal section, inverted fluorescence microscopy, ×40 magnification). (**i**) PKH26-labeled chondrocytes on scaffold after 1 week (7 d) (cross-section, inverted fluorescence microscopy, ×40 magnification). (**j**) PKH26-labeled chondrocytes on scaffold after 1 week (longitudinal section, inverted fluorescence microscopy, ×40 magnification). (**k**) Quantitative results of PKH26-labeled chondrocytes on the scaffolds. ** *p* < 0.01.

Chondrocyte composite scaffolds were intact and dull after culture for 1 week (7 d) in vitro, without any debris or fragmentation, shrinkage, or degradation. Samples gradually became translucent with a pink-white appearance (Figure 4a); the flexibility was obvious when forceps were used to clamp the compound, but the compound was not as firm as normal cartilage and could not be easily grasped and picked up. After the cell–scaffold complex was stained with Hoechst33258, the cells were arranged in a columnar and uniform manner along the tube of the stent (Figure 4b). AO-PI staining indicated that population viability exceeded 95% (Figure 4c,d). Additionally, chondrocytes could adhere to and grow well on the scaffold, and examination using inverted microscopy revealed that most of the cells were spherical. The cells were spherical and attached to the pores of the stent after being inoculated on the scaffold for 2 h (Figure 4e). After overnight culture, the cells grew and adhered along the stent tube (Figure 4f).

The labeled chondrocytes showed red fluorescence by inverted fluorescence microscopy, and their activity was not affected by the PKH26 dye. In scaffolds examined after 1 d, based on inverted fluorescence microscopy, the red chondrocytes were arranged horizontally around the gap of the scaffold, vertically along the tube in a columnar arrangement, and were evenly distributed inside the scaffold (Figure 3g,h). The fluorescence intensity increased after 1 week (7 d), indicating that the cells grew rapidly and secreted a large amount of matrix (Figure 4i,j). Quantitative analysis showed that the number of PKH26-labeled chondrocytes on the scaffold cultured for 1 week was significantly higher than that for 1 d (*p* < 0.01; Figure 4k), regardless of cross section or longitudinal section.

**Figure 5:**
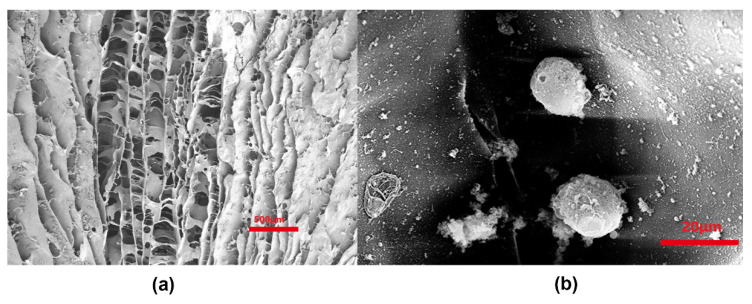
(**a**) Chondrocytes cultured for 7 d in vitro on scaffolds (scanning electron microscopy [SEM], ×200 magnification). (**b**) Cells had adhered to the material surface and secreted a large amount of filamentous matrix (SEM, ×500 magnification).

SEM observation of cells cultured on the scaffold overnight in vitro showed that the surface of the scaffold and the cells in the void were still round and arranged in a tubular column (Figure 5a). The secretion of ECM was apparent. The ECM adhered well to the surface of the scaffold and was widely distributed inside the scaffold (Figure 5b).

**Figure 6:**
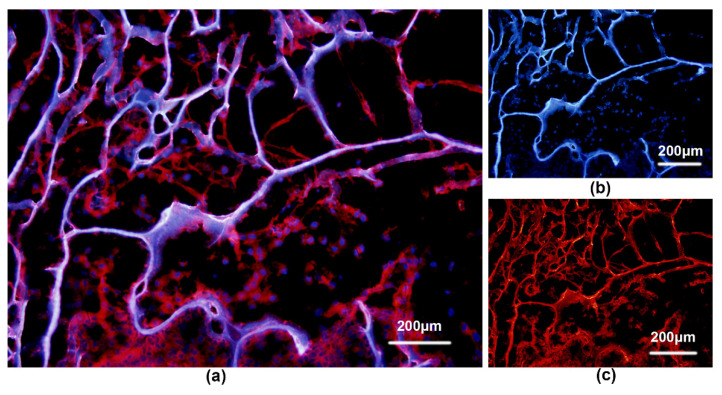
(**a**) Overlap of the two fields above reveals the large amount of collagen type II matrix secreted around the cells. (**b**) Hoechst33258 staining of a cell–scaffold complex revealing a large number of cells inside the scaffold. (**c**) Cell–scaffold complex (inoculated 3 d) examined by collagen type II immunofluorescence staining and fluorescence microscopy.

Hoechst33258 staining of cell–scaffold complexes showed the presence of cells inside the scaffold (Figure 6b). Red fluorescence indicated positive immunohistochemical staining with collagen type II (Figure 6c). Cells with overlapping fields of vision displayed pink fluorescence (Figure 6a).

### 3.5. Immunoreaction of ACECM Scaffolds

**Figure 7:**
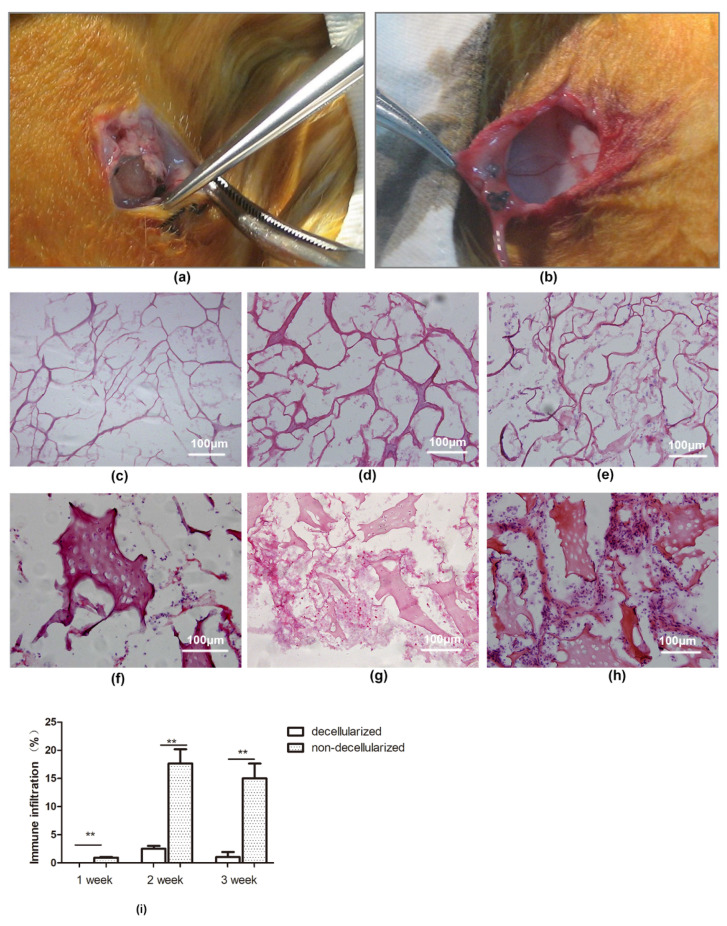
(**a**,**b**) Immune response assessment of the cartilage transplantation at the incision site after operation in rabbit. Porcine decellularized and non-decellularized cartilage scaffolds at 1 week after surgery (**c**,**f**); 2 weeks after surgery (**d**,**g**); and 4 weeks after surgery (**e**,**h**) (HE, original magnification × 200). (**i**) Quantitative results of the infiltrated cells in decellularized and non-decellularized cartilage scaffolds. ** *p* < 0.01.

Two groups of New Zealand white rabbits had no adverse reactions after implantation of allogeneic decellularized cartilage scaffolds and non-decellularized cartilage scaffolds, respectively. There was no obvious swelling, exudation and other inflammatory reactions at the incision site 1, 2 and 4 weeks after operation (Figure 7a,b). The wound healing was good, and the decellularized cartilage scaffold did not adhere to the surrounding tissues. Moreover, no capsule formation and vascular invasion were observed around the scaffold, and it was easy to separate the scaffold from the surrounding tissues.

**Figure 8:**
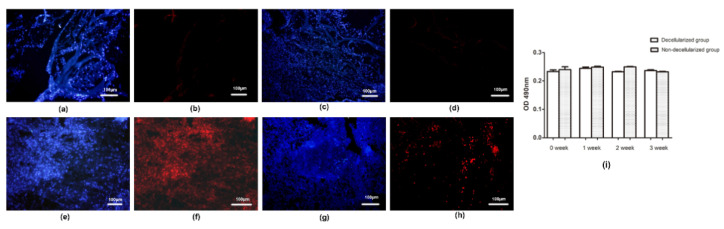
Immunohistochemical staining (×200). After the porcine decellularized scaffold was implanted in rabbits for 2 weeks (**a**–**d**) and 4 weeks (**e**–**h**), the scaffold and surrounding tissues were fluorescently labeled with CD4+\CD8+ lymphocyte subsets. The blue bright spot showed the nucleus, the red spot in (**b**,**f**) showed the presence of CD4+ lymphocytes, and the red spot in (**d**,**h**) showed the presence of CD8+ lymphocytes. (**i**) Quantitative results of the CD4+\CD8+ lymphocyte subsets in decellularized and non-decellularized cartilage scaffolds.

As illustrated in Figure 7c, there was no obvious cell infiltration in the decellularized cartilage scaffold 1 week after surgery, but a small number of prokaryotic cells infiltrated the non-decellularized cartilage scaffold (Figure 7f). 2 weeks after the operation, several cell infiltration was observed in the decellularized cartilage scaffold (Figure 7d), which was dominated by neutrophils, scattered with several CD4+T lymphocytes and CD8+T lymphocytes (Figure 8b,d). The number of infiltrated prokaryotic cells in the non-decellularized cartilage scaffolds at 2 weeks increased compared with 1 week after surgery (Figure 7g), and a small number of lymphocytes were observed. At 4 weeks postoperatie, the decellularized cartilage scaffold was partially degraded and the infiltrated cells were reduced (Figure 7e). Furthermore, there were still many prokaryotic cells infiltrated in the non-decellularized cartilage scaffold (Figure 7h), and quantitative analysis showed that the number of infiltrated cells in decellularized scaffold was significantly higher than that in non-decellularized cartilage scaffold at 1 week, 2 weeks and 3 weeks, respectively (all, *p* < 0.01; Figure 7i). Moreover, a large number of lymphocytes infiltrated in the scaffold and its surrounding tissues (Figure 8f,h), and no significant difference was found between decellularized and non-decellularized cartilage scaffold (*p* > 0.05; Figure 8i).

In addition, the results of humoral immunity showed that the antibody levels of the xenoacellular cartilage scaffolds did not increase at 1, 2 and 4 weeks after implantation compared with the preoperative antibody levels (*p* > 0.05). Similarly, there was no significant difference in the antibody level of the non-decellularized scaffolds at 1, 2 and 4 weeks after implantation compared with the preoperative antibody level, indicating that the implanted xenoacellular cartilage scaffolds did not induce antibody immune response.

## 4. Discussion

Low cell density and the absence of vasculature in AC leads to poor self-repair ability [23]. In this study, we successfully prepared AC decellularized scaffolds, cultured cartilage cells in vitro, and constructed cartilage cells and ACECM scaffold complexes in vitro. In addition, analysis of immunoreaction of ACECM scaffolds, such as inflammatory cell detection, cellular immune response assay and humoral immune response assay was conducted based on the heterogenous subcutaneous implantation of ACECM scaffolds. The results showed that chondrocytes on the scaffolds performed well in terms of adhesion and growth and secreted collagen type II moreover, the implanted xenoacellular cartilage scaffolds did not induce antibody immune response, suggesting that porcine ACECM-oriented scaffolding is an ideal natural biomaterial for cartilage tissue engineering.

Cartilage injury can cause joint diseases and affect joint functions in human. In recent years, with the advent of cartilage tissue engineering, the study of cartilage cell culture in vitro has gained increasing attention. However, most of the initial studies were based on cartilage pathology and drug reactions [24,25]. Owing to its simple structure and single-cell tissue type, cartilage has proven to be the ideal target for tissue engineering [26]. Cartilage tissue consists of chondrocytes and ECM (including collagen, proteoglycan, hyaluronic acid, glycoprotein, and other components). Based on the content of different types of collagen in the cartilage matrix, the cartilage of the body can be divided into three types, hyaline cartilage, fibrocartilage, and elastic cartilage. The collagen type I content is higher in the fibrocartilage matrix, the elastic fiber content is higher in the elastic cartilage matrix, and collagen type I is the main component of hyaline cartilage matrix although that is collagen type II. In the present study, the morphology of chondrocytes cultured in vitro was consistent with that of normal chondrocytes, indicating the success of this method in cell culture. Additionally, in vitro observation of the cell–scaffold complex revealed that the ECM scaffold of AC was beneficial for the adhesion, proliferation, and distribution of cartilage cells, which was significant for the construction of functional tissue-engineered cartilage.

The purpose of fabricating tissue-engineered cartilage scaffold materials is to provide a three-dimensional structure for cartilage cells to facilitate the adhesion and proliferation of cells and to provide a suitable environment for cell growth [27]. Given the unpredictable degradation of high-polymer synthetic material, inflammation caused by degradation products, and other events, many researchers have studied natural materials, such as alginate [28], collagen [29], fibrin [30], chitosan [31], and hyaluronan derivatives [32,33], with the goal of constructing scaffolds that could retain cartilage cells. AC decellularized scaffolds are similar to natural cartilage and might be an ideal natural biomaterial for clinical applications. Tissue-engineered cartilage constructed with an acellular cartilage scaffold is a promising option [34]. In our study, after 1 week (7 d) of culturing chondrocytes and ACECM scaffolds, the cells were attached to the scaffold wall and there was a large amount of cartilage inside the scaffold, indicating that chondrocytes inoculated into ACECM scaffolds could continue to grow and differentiate. Moreover, the immunohistochemical staining for collagen type II, as well as safranin “O” and toluidine blue staining, was positive, proving that chondrocytes could continue to grow and differentiate when inoculated into ACECM scaffolds. SEM revealed that the cells were spherical or elliptical and were surrounded and partially wrapped by a secreted matrix. Morphological observation confirmed the cytocompatibility of ACECM scaffolds, the characteristic morphological changes of the chondrocytes, and the secretion of ECM by the chondrocytes. This acellular method could completely remove the chondrocytes from the porcine AC and retain the main components of the ACECM, similar to the columnar arrangement of natural AC. All results revealed that ECM-derived scaffolds could enhance the biomechanical property of chondrocytes.

Chondrocytes as seed cells for constructing tissue-engineered cartilage conform with the physiological state and are the first choice for experimental research and clinical applications [35]. The in vitro expansion of autologous chondrocytes for the repair of full-thickness cartilage defects of the knee and ankle joints has been used in clinical practice and has been approved by the United States Food and Drug Administration with satisfactory clinical results [36]. However, autologous chondrocytes are associated with limited sources, are difficult to separate, proliferate slowly, and easily dedifferentiate during in vitro culture. These shortcomings limit their clinical development. In contrast, allogeneic chondrocytes are relatively sufficient.

Biomaterials can cause immune reactions [37,38], including inflammation, immunosuppression, immune stimulation, hypersensitivity and autoimmunity. Different biomaterials produce different cellular immune responses when they come into contact with blood or tissue. Hence, the study of immune response of biomaterials is of great significance for its own safety evaluation. In this study, CD4+ and CD8+T lymphocytes of the scaffold and surrounding tissues were tested in the decellularized cartilage scaffolds 1, 2 and 4 weeks after implantation. And there was no lymphocyte infiltration in the xenogenated decellularized cartilage scaffolds, while obvious lymphocyte infiltration was found in the non-decellularized cartilage scaffolds. Moreover, there was no significant difference in the antibody level of the non-decellularized scaffolds at 1, 2 and 4 weeks after implantation compared with the preoperative antibody level, indicating porcine ACECM scaffolds were used for xenotransplantation without significant immune inflammatory response. Taken together, all these findings suggested the immunogenicity of allogeneic decellularized cartilage scaffolds is very low, so it has potential clinical application. In addition, the repair effect of decellularized cartilage scaffolds on joint defects needs more attention. Therefore, the use of decellularized cartilage scaffolds for the repair of joint defects in homogeneous or heterogeneous animals needs further research.

## 5. Conclusions

In summary, the AC of miniature pigs was isolated and cultured, and phenotypic identification of chondrocytes was carried out. The experiments performed in this study provide an experimental basis for the use of chondrocytes in cartilage tissue engineering. Overall, these results indicate that ECM-derived scaffolds enhance the biomechanical property of chondrocytes and thus might serve as a useful approach to cartilage tissue engineering.

## Data Availability

Not applicable.

## Supplementary Materials

The following supporting information can be downloaded at: https://www.mdpi.com/article/10.3390/polym14142848/s1, Figure S1: The surgical procedure. (a) Anesthetic injection. (b) The skin and subcutaneous tissue were cut. (c) The cartilage scaffold was embedded between the deep fascia and sarcosis. (d) The skin incisions were sutured with 3-0 fine silk thread; Figure S2: (a) The survival rate of primary chondrocytes was observed with a fluorescence microscope with acridine orange-propidium iodide (AO-PI) staining. (b) Primary chondrocytes expanded and adhered to the wall. (c) Primary chondrocytes spread out on the wall attached to gauze. (d) Primary chondrocytes spread out on the wall attached to gauze. Figure S3: (a) First-generation chondrocytes. (b) Third-generation chondrocytes. (c) Positive chondrocyte slides stained with safranin O. (d) Chondrocyte type collagen II staining was positive. (e) Cartilage cells were positively stained with toluidine blue.
