# Supplementary material for: Fabrication of Tissue-Engineered Cartilage Using Decellularized Scaffolds and Chondrocytes
Source: Polymers (Basel). 2022 Jul 13;14(14):2848. doi: 10.3390/polym14142848 (PMC9316171; doi:10.3390/polym14142848)
Supplement: Supplementary file 1 [file polymers-14-02848-s001.zip › polymers-1751100-supplementary.pdf]

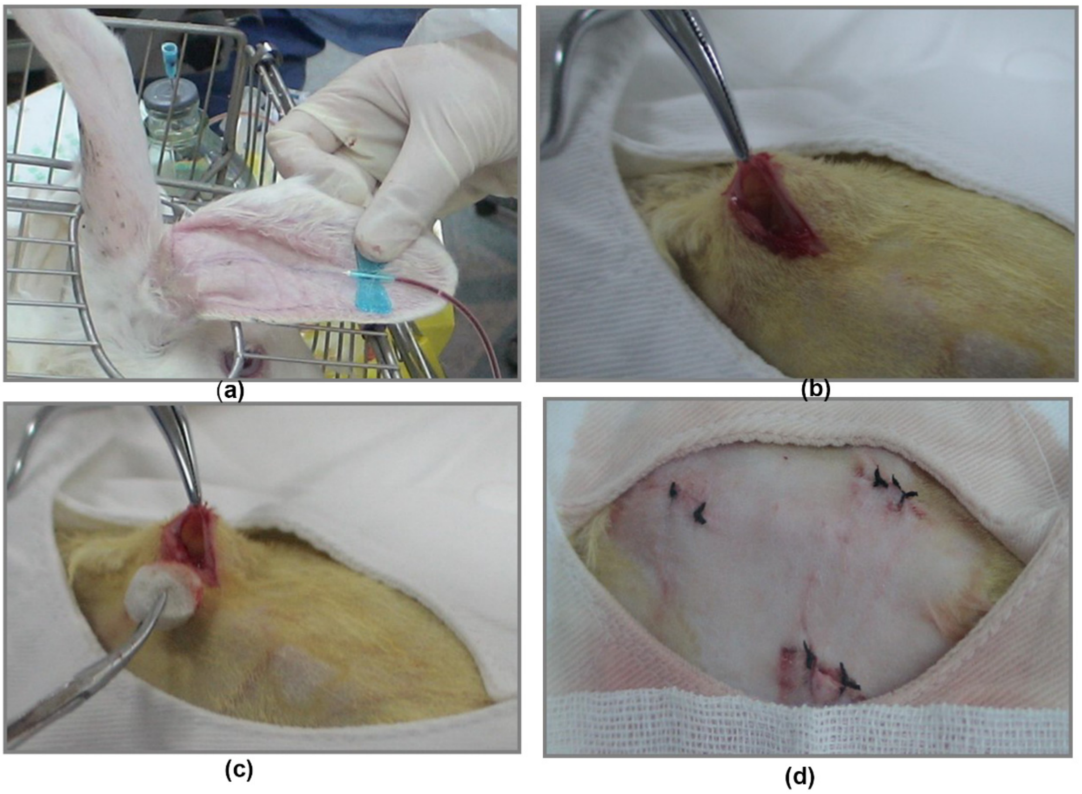

Figure S1 The surgical procedure. (**a**)Anesthetic injection. (**b**) The skin and subcutaneous tissue were cut. (**c**) The cartilage scaffold was embedded between the deep fascia and sarcosis. (**d**) The skin incisions were sutured with 3-0 fine silk thread.

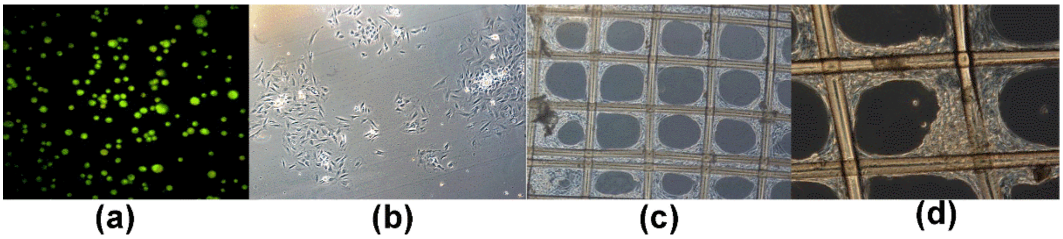

Figure S2 (**a**) The survival rate of primary chondrocytes was observed with a fluorescence microscope with acridine orange-propidium iodide (AO-PI) staining. (**b**) Primary chondrocytes expanded and adhered to the wall. (**c**) Primary chondrocytes spread out on the wall attached to gauze. (**d**) Primary chondrocytes spread out on the wall attached to gauze.

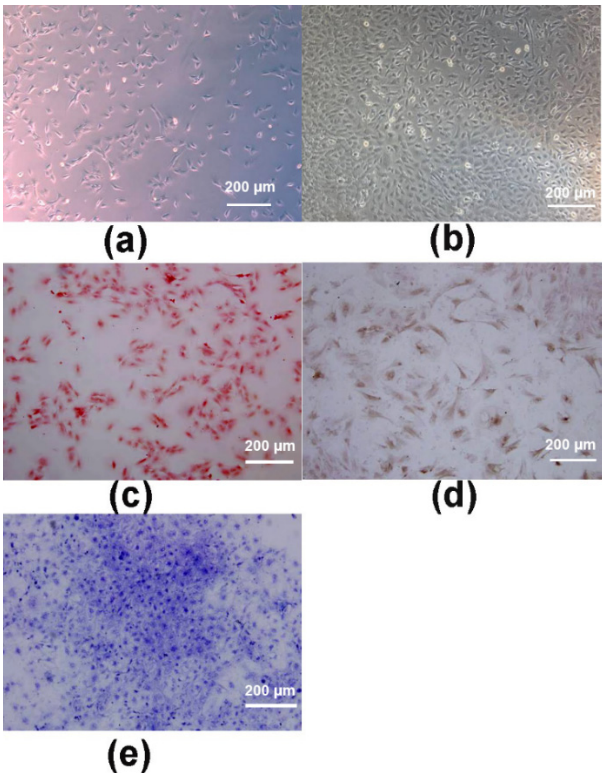

Figure S3 (**a**) First-generation chondrocytes. (**b**) Third-generation chondrocytes. (**c**) Positive chondrocyte slides stained with safranin O. (**d**) Chondrocyte type collagen II staining was positive. (**e**) Cartilage cells were positively stained with toluidine blue.
